# The relationship between death anxiety and self-esteem: A protocol for a systematic review and meta-analysis

**DOI:** 10.1371/journal.pone.0346665

**Published:** 2026-04-03

**Authors:** Tadgh Connery, Sofija Kukulite, Conor Farrell, Rosa Horgan, Karen Barry, Megan Doyle, Annalisa Setti, Mike Murphy

**Affiliations:** 1 School of Applied Psychology, University College Cork, Cork, Ireland; 2 Environmental Research Institute, University College Cork, Cork, Ireland; King Khalid University, EGYPT

## Abstract

**Background:**

Death anxiety is fast becoming recognised as a transdiagnostic construct across myriad mental health conditions. Though existing clinical treatments of death anxiety, such as Cognitive Behaviour Therapy type interventions, have been shown to be effective, Terror Management Theory (TMT) proposes self-esteem as a protective factor against death anxiety by endowing individuals with a feeling of significance in life and helping them to process the nature of death and its inevitability. Despite the centrality of self-esteem to TMT, however, extant research examining its relationship with death anxiety has yet to be synthesised. The current study aims to systematically review peer-reviewed, quantitative research examining the association between death anxiety and self-esteem and, where possible, to test the strength of these associations through meta-analysis.

**Method:**

A systematic search of quantitative and mixed-methods studies will be conducted across six databases: MEDLINE; PsycINFO; PubMed; Web of Science; CINAHL and; EMBASE. Google Scholar will also be searched and the first 200 records by relevance will be screened for eligibility. Searches will be conducted by TC, and records will be screened by TC, SK, CF, RH and KB, so that each record is screened for eligibility by at least two authors. Data extraction and quality assessment, using the Joanna Briggs Inventory Risk of Bias tools, will be performed by TC, SK, CF, RH and KB, so that each record is assessed by at least two authors, with doubts and discrepancies being resolved through discussion with AS and MM. A narrative synthesis of relevant data will be presented and, where sufficient data are available, meta-analysis will be conducted using the MAJOR extension for Jamovi to establish an overall effect size for the association between death anxiety and self-esteem. Should sufficient data be available, demographic factors, such as gender and age, and clinical population status (clinical vs. non-clinical population) will be examined as moderators of the effect. This protocol was developed in line with the Preferred Reporting Items for Systematic Reviews and Meta-Analyses (PRISMA) guidelines and has been registered in PROSPERO (CRD42024591775).

**Discussion:**

The current review will systematically examine the relationship between death anxiety and self-esteem. Should sufficient data be available, an overall effect size of the relationship will be generated. By better understanding how death anxiety is related to self-esteem, and by establishing the size and significance of the relationship will aid firstly in systematically validating TMT and, secondly, identify whether self-esteem may be targeted in future death anxiety interventions to reduce death anxiety and improve individuals’ overall mental health.

## Introduction

Though all organisms die, humans are unique in their awareness of mortality and their ability to reflect and ascribe meaning to death [1]. Despite this, humans possess a drive for self-preservation and live their lives in ways to prolong death for as long as possible [2]. The biological predisposition of humans for self-preservation, coupled with an awareness of mortality, generates a psychological conflict that leads to the emergence of death anxiety [3]. Death anxiety refers to by the fear of one’s own and others’ death, the apprehension of death’s inevitability, and the uncertainty about post-death existence [4,5].

Death anxiety is fast becoming recognised as a transdiagnostic determinant of multiple psychopathologies [6], with positive associations being identified between death anxiety and poor mental health outcomes such as peritraumatic stress [7], obsessive-compulsive disorder [8], fear of illness recurrence or progression [9] and specific phobias [10,11]. A recent meta-analysis of 99 cross-sectional studies reported a moderate, positive effect size between death anxiety and mental health symptoms (r = .397), with larger effects observed in clinical samples (r = 0.58) compared to non-clinical samples (r = 0.331) [6]. Among these, anxiety-related disorders were seen to correlate strongly with death anxiety (r = 0.68), while obsessive-compulsive symptoms demonstrated a moderate correlation with death anxiety (r = 0.48) [6].

If left unaddressed, death anxiety may persist and potentially manifest into other mental health disorders [6], illustrating the revolving door phenomenon whereby individuals return to a service with a new condition due to inadequate treatment. This underscores the necessity of clinical interventions, such as Cognitive Behavioural Therapy (CBT), which target death anxiety directly. A meta-analysis examining the impact of 15 randomised controlled trials examining the effectiveness of death anxiety interventions found CBT-based interventions and, in particular, those that focus on deliberate and systematic exposure to death-related stimuli to be the most effective in reducing death anxiety [12]. In a Phase I trial of an online CBT-based intervention, 60% of 20 clinical participants demonstrated decreased death anxiety overall, with 90% experiencing a decrease on at least one facet of death anxiety, pointing to its effectiveness in treating death anxiety in clinical samples [5].

While CBT seems most effective in reducing death anxiety [12], other psychological interventions have also demonstrated effectiveness in reducing death anxiety. For instance, one systematic review of nine studies examining the effects of death anxiety interventions for 1,179 cancer patients found that interventions centred around meaning, dignity, relationships and spiritual wellbeing were associated with reductions in death anxiety [13]. Similarly, a study involving 50 cancer patients demonstrated that a brief individual psychotherapy which addressed psychological and existential distress associated with advanced illness, resulted in a significant reduction in death anxiety [14]. Thus, while CBT-based interventions present effective reductions in death anxiety, other psychological interventions also demonstrate notable benefits.

Terror Management Theory (TMT), the leading social psychology theory explaining death anxiety, is rooted in the conviction that self-esteem and cultural worldviews serve as essential buffers against the development of death anxiety [15]. This process emphasises the establishment of symbolic immortality, which subsequently diminishes the likelihood of death anxiety [1]. Literal or symbolic immortality can be attained through participation in cultural worldviews, which offer a sense of meaning and purpose to one’s life, easing individual’s death-related concerns [5,15,16]. Meeting or exceeding expectations within cultural worldviews can increase one’s self-esteem and allow individuals to view themselves positively and as of value within the group [15,17]. According to TMT, individuals with higher self-esteem view themselves as worthwhile and significant, which allows them to process the nature of death and its inevitability, and protects them against death anxiety [1]. Meta-analysis of 164 mortality salience studies concluded that when self-esteem was enhanced through experimental manipulation, individuals felt more secure in their self-worth, and as a result, did not rely on cultural worldviews to manage their fear of death [18], reiterating TMT’s proposition that self-esteem buffers death anxiety.

Though TMT continues to be a leading and empirically supported theory within death anxiety research, a systematic overview of the association between death anxiety and self-esteem is absent from the current literature. Due to the significant effects of death anxiety on mental health, understanding how death anxiety and self-esteem are related will aid in informing the development of appropriate psychological interventions, such as reminiscence-based interventions [19] aimed at enhancing self-esteem to subsequently reduce death anxiety. Given the centrality of self-esteem to TMT and its proposed role as a death anxiety buffer, there is potential that self-esteem-based interventions may reduce death anxiety or, indeed, protect individuals against developing debilitating levels of death anxiety. Clarifying the nature and strength of its association with death anxiety will not only help in understanding the extent to which self-esteem holds potential as a modifiable protective factor against death anxiety, but it will also aid in systematically evaluating the validity of TMT.

### Aim of the review

The primary aim of this review is to address the gaps in the current literature by narratively synthesising existing research examining associations between death anxiety and self-esteem. Where sufficient data are available, a meta-analysis will be conducted to ascertain an overall effect size of this association.

The proposed review will answer the following review questions:

1. In adult participants (populations), to what extent is self-esteem (exposure) associated with death anxiety (outcome)?
2. Do demographic factors, such as gender or age, moderate the relationship between death anxiety and self-esteem?

### Objectives

1. The primary objective is to narratively synthesise existing research examining associations between death anxiety and self-esteem, and to test the strength of these associations using meta-analysis, where sufficient data are available.
2. The secondary objective, where sufficient data are available, is to examine whether demographic factors, such as gender and age, moderate the associations between death anxiety and self-esteem.

### Methods and analysis

Adherence to the Preferred Reporting Items for Systematic Reviews and Meta-Analyses Protocols (PRISMA-P) guidelines shaped the development of this protocol [20,21]. The protocol was registered with the International Prospective Register of Systematic Reviews (PROSPERO; CRD42024591775). See supporting information for a completed PRISMA-Protocols checklist (S1 File).

### Types of studies

In developing inclusion and exclusion criteria for this systematic review protocol, the acronym PECO (Population, Exposure, Comparisons, Outcome) was employed (Table 1). Inclusion will be exclusively limited to peer-reviewed quantitative studies, including cross-sectional, experimental, quasi-experimental and intervention studies. To ensure that valuable sources of relevant quantitative data are not ignored, mixed-methods studies that concentrate on quantitative associations between death anxiety and self-esteem will be included, and only their relevant quantitative data will be extracted. For a quantitative or mixed-methods study to merit inclusion, it must report an effect size representing at least one of the following: 1. a correlation between death anxiety and self-esteem; 2. an effect size representing changes in self-esteem between pre-mortality salience and post-mortality salience timepoints; 3. an effect size representing differences in post-mortality salience death-thought accessibility across participants with high and low self-esteem, or; 4. Data from a TMT-based experimental study where self-esteem is included either an independent, dependent or moderator variable.

**Table 1:** Eligibility criteria for the systematic review and meta-analysis.

PECO Acronym	Inclusion criteria	Exclusion criteria
P	Adult participants, aged 18 years and above	Participants under the age of 18 years
E	Measure of self-esteem	No measure of self-esteem
C	Not applicable	Not applicable
O	Measure of death anxiety	No measure of death anxiety
Additional criteria	Quantitative studies, including correlational, experimental, quasi-experimental and intervention studies Mixed-methods studies (only quantitative data will be extracted)	Qualitative studies, policy briefs or reports, opinion papers, commentaries, editorials, news, theoretical papers, conference presentations, dissertations, literature reviews, systematic reviews and meta-analyses

Qualitative studies, policy briefs or reports, opinion papers, commentaries, editorials, news, theoretical papers, conference presentations, dissertations, literature reviews, systematic reviews and meta-analyses will be excluded. In an effort to reduce risk of language bias, no language restriction will be used in determining exclusion of records [22]. Should non-English language records be available in languages spoken by members of the research team (Italian, French, Spanish, Latvian), they will be translated by the respective team member. Otherwise, non-English language records will be translated using a digital translation tool to translate the record to English [22].

### Types of participants

The review will include studies that recruit adult participants (aged 18 years and above (population), for whom measures of self-esteem (exposure) and death anxiety (outcome) are reported.

### Patient and public involvement

Given that the current research examines previously published data, participants were not directly involved or recruited for this review, and patient and public involvement was not necessary.

### Types of outcome measures

The primary outcome measures for this review will be death anxiety and self-esteem, each measured by a validated scale in adult participants (aged 18 and above). Studies will only be included if they include a measure of effect for an association between death anxiety and self-esteem. Correlational research will be included if a correlation coefficient denoting the association between death anxiety and self-esteem is reported. Experimental studies (i.e., mortality salience studies) will be included if they examine changes in self-esteem between pre-mortality salience and post-mortality salience timepoints, or if they examine differences in post-mortality salience death-thought accessibility across participants with high and low self-esteem. TMT-based experimental studies will also be included, so long as self-esteem is included in the study design as either an independent variable or moderator variable.

### Measures of effect

It is likely that some variety in scores and outcomes identified will be present, given the diversity of measurement scales. Thus, the frequency and prevalence of measurement scales will be noted in the data extraction sheet. Means, standard deviations, effect sizes and p-values will be extracted as measures of effect.

### Search method

The search strategy was developed by TC and MD, in consultation with the UCC Academic Librarian. A systematic search of six academic databases (MEDLINE; PsycINFO; PubMed; Web of Science; CINAHL and; EMBASE) will be conducted using the following search string: (“death anxi*” OR “thanatophobia” OR “fear* of death” OR “fear* about death” OR “dying anxi*” OR “death attitudes” OR “death anxiety” OR “terror management” OR “terror management theory” OR “mortality salience” OR “death thought accessibility”) AND (“self esteem” OR “self-esteem” OR “self worth” OR “self concept” OR “self evaluation*” OR “self attitude*” OR “self liking” OR “self competen*” OR “self perception*”). Titles and abstracts will be searched for the search string terms by TC.

In addition to searching six academic databases, Google Scholar will also be searched, given its utility in supplementing searches of academic databases, with recommendations stating that on the first 200–300 records is optimal, as most sources that appear after the 200^th^ record are, on average, grey literature [23]. Adhering with these recommendations, Google Scholar will also be searched using the search string, and the first 200 records will be retrieved and screened for eligibility. TC will conduct these searches in December 2025.

### Study selection

Identified records will be independently screened by members of the research team (TC, SK, CF, RH, KB), first by title and abstract and then by full text, so that each record is screened by at least two authors at each stage. The screening process’ inter-rater reliability will be calculated using the kappa coefficient. A data sheet designed by TC will be used for the purpose of this study.

Data will be extracted using a data extraction sheet designed by TC for the purpose of the review. Five authors (TC, SK, CF, RH, KB) will independently extract relevant data, so that data from each record are extracted by at least two authors, with conflicts and discrepancies being resolved through discussion and consensus with AS and MM. A PRISMA flow diagram will document the screening process, including reasons for excluding records. Data extraction will be completed by the end of February 2026.

### Data extraction process

The following data will be extracted from included papers:

- Authors and publication year
- Region
- Sample description
- Study setting
- Measurement tools for death anxiety and self-esteem
- Effect sizes (e.g., correlations, F-values, chi-square values), p-values, means and SDs

### Missing data

If data or details pertaining to a study’s procedure are missing from the report, the relevant corresponding author/co-author will be contacted in an effort to retrieve them. Failing this, the data will be excluded from analysis, and this will be addressed in the review’s discussion section.

### Risk of bias assessment

The quality of evidence and methodology of included studies will be assessed using the Joanna Briggs Institute Critical Appraisal Tools. The appropriate risk of bias tool (i.e., Checklist for Analytical Cross-Sectional Studies, Checklist for Quasi-Experimental Studies, Checklist for Randomised Control Trials) will be applied to assess each included record’s quality based on its study design. Quality assessment will be conducted for each record independently by TC, SK, CF, RH and KB so that each included record is assessed by at least two authors. Papers will be classified as being low, medium and high risk, depending on their rating for each criterion. Each author’s assessments will be compared, and an inter-rater reliability coefficient will be calculated using the kappa coefficient. Doubts and discrepancies between the team will be resolved through mutual deliberation and consensus between the research team, AS and MM. Failing to reach consensus, MM will resolve the discrepancy as senior author.

### Data synthesis

A narrative synthesis will be conducted of the included papers examining associations between death anxiety and self-esteem, in accordance with Cochrane guidelines [24]. Sample characteristics will be described and measurement tools for both death anxiety and self-esteem will also be noted.

### Meta-analysis

For studies that present correlations between death anxiety and self-esteem, meta-analysis will be conducted with the pooled results using the MAJOR extension for Jamovi. Should sufficient data be available, demographic variables, such as gender and age, and clinical population status (clinical vs. non-clinical populations) will be examined as potential moderators of the relationship. Gender will be evaluated using data as reported within the individual included studies for the purpose of this review.

For non-correlational studies, relevant effect sizes (e.g., F-values, t-test effect sizes, chi-square values) will be extracted and standardised to allow for their inclusion in meta-analysis. Outcomes will be transformed into standardised mean difference effect sizes (Cohen’s d) and meta-analysis will be conducted to assess the weighted standardised mean difference across studies using Cohen’s d and standard error values.

I^2^ values will calculate the statistical heterogeneity between studies, with values <25% indicating low heterogeneity, values between 25–50% indicating moderate heterogeneity and values >50% indicating high heterogeneity. A random-effects model will be used for highly heterogeneous studies (>50%) and, where the level of heterogeneity is not significant, a fixed-effects model will be applied to perform data pooling. A forest plot will also be generated and visually inspected for heterogeneity. Variance in the position of each study’s confidence intervals may suggest high heterogeneity. Should high heterogeneity be observed, meta-regression will be run to examine if the effect size changes based on specific study characteristics and sub-group analysis will investigate whether these individual characteristics act as sources of heterogeneity.

Visual examination of the funnel plot, the trim and fill procedure [25] and Egger’s regression test will assess included studies’ publication bias. Publication bias may be indicated by asymmetry in the funnel plot or by a significant intercept (p < .05) in Egger’s regression test. The trim and fill procedure will allow for publication bias to be adjusted by identifying which studies contribute to funnel plot asymmetry in the funnel plot, removing them to recalculate a more symmetrical funnel plot and associated pooled effect size and by replacing the originally removed studies with imputed mirror counterparts to create an unbiased, symmetrical plot [25]. A revised estimated effect size will be reported using the trim and fill procedure, should publication bias be identified through these procedures.

## Discussion

This study aims to systematically review peer-reviewed, quantitative research that examines the associations between death anxiety and self-esteem. Where sufficient data are available, an overall effect size will be calculated for the association between death anxiety and self-esteem.

### Strengths and limitations of the study protocol

This review’s notable strength is its adherence to a PRISMA-compliant methodology, ensuring transparency, reproducibility, and efficiency. The Joanna Briggs Institute Checklist provides a standardised framework for assessing evidence quality and methodological rigour in this study. The allocation of tasks within the research team will enhance efficiency [26], in turn reducing redundancy in efforts within the research team. A further strength of this review is its inclusion of both correlational and experimental studies, facilitating the examination of associations between variables [27] and the identification of potential cause-and-effect relationships [28]. This will broaden the scope of evidence and enhance our understanding of the associations between death anxiety and self-esteem. The inclusion of non-English language articles is also a strength, as it will help to reduce language bias within the current review [22] and allow for an identification of the scope of potential cultural variability in research examining death anxiety and self-esteem. Additionally, the measurement of death anxiety and self-esteem may vary across studies, potentially limiting comparability and reducing the precision of meta-analytical conclusions. Further, the inclusion of cross-sectional studies in the current review will limit inferences around causality between death anxiety and self-esteem.

### Implications of the study protocol

Though self-esteem is central to TMT, a lack of a systematic overview of the association between death anxiety and self-esteem has limited our understanding of this relationship. Systematically evaluating the relationship between death anxiety and self-esteem will also aid in systematically evaluating the validity of TMT.

The current study also holds clinical implications. Systematically evaluating whether a significant relationship exists between death anxiety and self-esteem may clarify the role of self-esteem in treating death anxiety. Should a significant negative relationship be found, the findings will inform the development of death anxiety interventions and may point to the use of self-esteem interventions, which would offer an alternative to already-validated death anxiety interventions such as CBT [12] and meaning-centred interventions [13]. While existing CBT-type interventions have been effective in reducing death anxiety specifically, self-esteem interventions may aid in bolstering individuals’ resilience against death anxiety, lowering their susceptibility to death anxiety and subsequent need for clinical intervention. Subject to data availability, examining whether demographic factors, such as gender and age, moderate the effect between death anxiety and self-esteem will allow for an identification as to who might particularly benefit from self-esteem interventions, in terms of death anxiety, and subsequently aid in promoting better global mental health.

## Supporting information

S1 File S1_prsimap_checklist_completed. (DOCX)

## References

[1] Pyszczynski T, Solomon S, Greenberg J. Thirty years of terror management theory: From genesis to revelation. Advances in experimental social psychology. Academic Press. 2015. p. 1–70.

[2] Jong J. Death anxiety and religion. Curr Opin Psychol. 2021;40:40–4. doi: 10.1016/j.copsyc.2020.08.004 32942111

[3] Kesebir P. A quiet ego quiets death anxiety: humility as an existential anxiety buffer. J Pers Soc Psychol. 2014;106(4):610–23. doi: 10.1037/a0035814 24660992

[4] Barrett C. Death Anxiety. Encyclopedia of Behavioral Medicine. Springer International Publishing; 2020. 603–4. doi: 10.1007/978-3-030-39903-0_1580

[5] Menzies RE, Menzies RG. Death anxiety and mental health: Requiem for a dreamer. J Behav Ther Exp Psychiatry. 2023;78:101807. doi: 10.1016/j.jbtep.2022.101807 36435549

[6] Menzies RE, McMullen K, Riotto GD, Iliescu S, Petrovic B, Remfrey M. From dread to disorder: A meta-analysis of the impact of death anxiety on mental illness symptoms. Clin Psychol Rev. 2024;113:102490. doi: 10.1016/j.cpr.2024.102490 39208495

[7] Siegel A, Mor I, Lahav Y. Profiles in COVID-19: peritraumatic stress symptoms and their relation with death anxiety, anxiety sensitivity, and emotion dysregulation. Eur J Psychotraumatol. 2021;12(1):1968597. doi: 10.1080/20008198.2021.1968597 34589177 PMC8475101

[8] Menzies RE, Zuccala M, Sharpe L, Dar-Nimrod I. Subtypes of obsessive-compulsive disorder and their relationship to death anxiety. Journal of Obsessive-Compulsive and Related Disorders. 2020;27:100572. doi: 10.1016/j.jocrd.2020.100572

[9] Menzies RE, Connery T, Macdonald D, Riotto GD. The relationship between death anxiety and fear of recurrence and progression in chronic illness: A systematic review and meta-analysis. J Psychosom Res. 2025;198:112373. doi: 10.1016/j.jpsychores.2025.112373 40974620

[10] de Jong PJ, Merckelbach H. Blood-injection-injury phobia and fear of spiders: Domain specific individual differences in disgust sensitivity. Personality and Individual Differences. 1998;24(2):153–8. doi: 10.1016/s0191-8869(97)00178-5

[11] Menzies RE, Sharpe L, Dar-Nimrod I. The relationship between death anxiety and severity of mental illnesses. Br J Clin Psychol. 2019;58(4):452–67. doi: 10.1111/bjc.12229 31318066

[12] Menzies RE, Zuccala M, Sharpe L, Dar-Nimrod I. The effects of psychosocial interventions on death anxiety: A meta-analysis and systematic review of randomised controlled trials. J Anxiety Disord. 2018;59:64–73. doi: 10.1016/j.janxdis.2018.09.004 30308474

[13] Grossman CH, Brooker J, Michael N, Kissane D. Death anxiety interventions in patients with advanced cancer: A systematic review. Palliat Med. 2018;32(1):172–84. doi: 10.1177/0269216317722123 28786328

[14] Lo C, Hales S, Jung J, Chiu A, Panday T, Rydall A, et al. Managing Cancer And Living Meaningfully (CALM): phase 2 trial of a brief individual psychotherapy for patients with advanced cancer. Palliat Med. 2014;28(3):234–42. doi: 10.1177/0269216313507757 24170718

[15] Greenberg J, Pyszczynski T, Solomon S. The Causes and consequences of a need for self-esteem: a terror management theory. Public Self and Private Self. Springer New York. 1986. p. 189–212. doi: 10.1007/978-1-4613-9564-5_10

[16] Bassett JF, Bussard ML. Examining the complex relation among religion, morality, and death anxiety: religion can be a source of comfort and concern regarding fears of death. Omega (Westport). 2021;82(3):467–87. doi: 10.1177/0030222818819343 30572785

[17] Lifshin U, Horner DE, Helm PJ, Solomon S, Greenberg J. Self-esteem and immortality: Evidence regarding the terror management hypothesis that high self-esteem is associated with a stronger sense of symbolic immortality. Personality and Individual Differences. 2021;175:110712. doi: 10.1016/j.paid.2021.110712

[18] Burke BL, Martens A, Faucher EH. Two decades of terror management theory: a meta-analysis of mortality salience research. Pers Soc Psychol Rev. 2010;14(2):155–95. doi: 10.1177/1088868309352321 20097885

[19] Niveau N, New B, Beaudoin M. Self-esteem Interventions in Adults – A Systematic Review and Meta-analysis. Journal of Research in Personality. 2021;94:104131. doi: 10.1016/j.jrp.2021.104131

[20] Moher D, Shamseer L, Clarke M, Ghersi D, Liberati A, Petticrew M, et al. Preferred reporting items for systematic review and meta-analysis protocols (PRISMA-P) 2015 statement. Syst Rev. 2015;4(1):1. doi: 10.1186/2046-4053-4-1 25554246 PMC4320440

[21] Shamseer L, Moher D, Clarke M, Ghersi D, Liberati A, Petticrew M, et al. Preferred reporting items for systematic review and meta-analysis protocols (PRISMA-P) 2015: elaboration and explanation. BMJ. 2015;350:g7647. doi: 10.1136/bmj.g7647 25555855

[22] Mheissen S, Spineli LM, Daraqel B, Alsafadi AS. Language bias in orthodontic systematic reviews: A meta-epidemiological study. PLoS One. 2024;19(4):e0300881. doi: 10.1371/journal.pone.0300881 38557691 PMC10984547

[23] Haddaway NR, Collins AM, Coughlin D, Kirk S. The role of google scholar in evidence reviews and its applicability to grey literature searching. PLoS One. 2015;10(9):e0138237. doi: 10.1371/journal.pone.0138237 26379270 PMC4574933

[24] Ryan R. Cochrane Consumers and Communication Review Group: Data Synthesis and Analysis. Cochrane Consumers and Communication Review Group. 2013. http://cccrg.cochrane.org

[25] Duval S, Tweedie R. Trim and fill: A simple funnel-plot-based method of testing and adjusting for publication bias in meta-analysis. Biometrics. 2000;56(2):455–63. doi: 10.1111/j.0006-341x.2000.00455.x 10877304

[26] Pechová J, Volfová H, Jírová A. Impact of task assignment on effectiveness in work teams. JESI. 2023;10(4):152–70. doi: 10.9770/jesi.2023.10.4(10

[27] Putri L, Rezani MR, Hermina D. Correlational research design. Jurnal Riset Multidisiplin Edukasi. 2025;2(6):306–17. doi: 10.71282/jurmie.v2i6.456

[28] Em S. Exploring experimental research: methodologies, designs, and applications across disciplines. Designs, and Applications Across Disciplines; 2024.

